# A prospective study for evaluating the effect of gastric targeted biopsy sampling with I‐scan optical enhancement on the diagnostic yield of CLOtest for *Helicobacter pylori* infection

**DOI:** 10.1002/hsr2.621

**Published:** 2022-04-22

**Authors:** Hosam Mohamed Dawod

**Affiliations:** ^1^ Tropical Medicine Department, Faculty of Medicine Zagazig University Al Sharkia Egypt

**Keywords:** CLOtest, *H. pylori*, I‐scan OE, targeted biopsy

## Abstract

**Background and Aim of the Work:**

*Helicobacter pylori*gastritis can cause serious adverse effects in the short and long term. I‐scan optical enhancement (OE) has a potential role to distinguish areas of infected mucosa and allow for targeted biopsy. It improves visual contrast and mucosal pattern characterization. The work aims to determine if the diagnostic yield of the CLOtest could be improved by using endoscopic I‐scan OE technology for targeted gastric biopsy sampling.

**Patients and Methods:**

A prospective study recruited 112 adult patients with active *H. pylori* infection diagnosed by C^13^ UBT at Nizwa General Hospital from March 2021 to January 2022. The patients underwent a careful examination by nonmagnifying upper endoscopy and I‐scan OE 3 moods, then randomly allocated into two groups. Group A: nontargeted double biopsies from the antrum and mid corpus. Group B: I‐scan OE‐directed targeted biopsy from abnormal mucosal patterns. The biopsy specimens were inoculated into CLOtest kits; the reading time of the positive results was at 1, 4, and 24 h.

**Results:**

Group B had a 92.8% positive CLOtest compared to 89.3% in group A (*p* = 0.501). One‐hour CLOtest was positive in 78.5% of the patients in group B compared to 60.7% in group A (*p* = 0.047), while group A had a significantly more positive CLOtest at 24 h.

**Conclusion:**

Sampling a targeted gastric biopsy with the aid of I‐scan ‐OE for CLOtest significantly hastens the positive reading time with high sensitivity.

## 1 INTRODUCTION

The primary lesion of *Helicobacter pylori* infection is progressive mucosal inflammation which may lead to significant adverse outcomes like atrophic gastritis, intestinal metaplasia (IM), peptic ulcer disease (PUD), gastric cancer, MALT lymphoma.^1^ Diagnosis of *H. pylori* infection and subsequent eradication therapy will prevent the disease progression and decrease the cancer risk.^2^ The rapid urease test (RUT) is an invasive test that requires sampling of the gastric mucosa. It is commonly used in upper endoscopy to diagnose active *H. pylori* infection.^3^ There are several different commercially available RUT kits. The choice of RUT depends on the availability and local preference because none has proven to be superior.^4^ A positive RUT requires approximately a minimum bacterial load of 10^5^ *H. pylori* in the biopsy sample.^5^ The time the test turns positive depends on the concentration of bacteria.^4^ Most of the positive test readings occurred within 1–3 h, especially in areas with a high prevalence of *H. pylori* infection,^6^, ^7^ and only became reliable after 4 h when making a treatment decision.^8^ However, it is best to hold those that appear negative for 24 h to avoid false‐negative.^3^, ^8^ The sensitivity of various RUT tests varied from 85% to 95% and specificity from 95% to 99%.^1^, ^9^ Adding the number of biopsy specimens will increase the accuracy of RUT.^9^, ^10^ On the other side, the patchy and uneven distribution of *H. pylori* infection, especially after antibiotics or proton pump inhibitors (PPIs),^11^, ^12^ atrophic gastritis, and IM reduce the test sensitivity and may cause false‐negative results.^1^, ^9^ Two biopsies, one from the antrum on greater curvature and one from the normal‐appearing corpus, are considered to produce a higher yield than one from the antrum,^9^, ^11^, ^12^ as mucosal atrophy is more prevalent at the antrum with few *H. pylori* organisms.^1^

Several gastric mucosal findings have a high probability of harboring *H. pylori* infection and can be identified as a predictor or highly suggestive for *H. pylori* infection such as mucosal edema, diffuse homogenous redness, antral nodularity, and enlarged gastric mucosal folds.^13^, ^14^ A recent meta‐analysis validated the diagnostic accuracy of specific abnormal mucosal patterns (mucosal edema and diffuse homogenous redness). It concluded that they are significant endoscopic findings associated with *H. pylori* current infection.^15^ The combination of endoscopic abnormal mucosal patterns has more predictive value for *H. pylori* active infection sites.^16^ Kyoto global consensus reported that endoscopic assessment is a desirable method for increasing the diagnostic yield of targeted biopsies in *H. pylori* gastritis. It allows for mucosal identification for targeted biopsy sampling.^17^ Studies are needed to consider the role of targeted gastric biopsy to increase the yield of the RUT test (3). Therefore, endoscopy has a potential role to distinguish areas of infected mucosa and allow for targeted biopsy sampling for the presence of *H. pylori* organisms by RUT.^18^ Furthermore, multiple nontargeted biopsies usually add to the cost and time of the procedure, risk of infection, and maybe slipped, crushed, or small if combined during sampling. *H. pylori* infection may be missed if not present in random biopsy samples from normal‐looking mucosa with a resultant increase of false‐negative results.^19^

I‐scan technology is a digital, image‐enhanced endoscopic (IEE) technology by PENTAX Medical. It enhances the endoscopic detection, demarcation, and characterization of the mucosal and vascular patterns by providing a clear, enhanced image and detailed topography. It can detect more subtle mucosal abnormalities,^17^, ^20^ and it is easy to observe and assess the abnormal mucosa with the aid of I‐scan technology and gain more information from visual examination of the gastric mucosa than standard white light endoscopy (WLE).^18^, ^19^, ^21^ The nonmagnifying I‐scan exam provides better image quality of *H. pylori* gastritis.^22^ It is superior to the WLE in identifying *H. pylori* infection‐related abnormal endoscopic features with higher diagnostic accuracy.^23^

Pentax's new endoscopic platform integrated the second‐generation “I‐scan optical enhancement” (OE). It incorporates an optical filter with improved visual contrast and provides extra information through improving vessel and mucosal pattern characterization.^17^ The work aimed to determine if the diagnostic yield of CLOtest can be improved by using I‐scan OE technology for targeted biopsy sampling from areas with endoscopic abnormal mucosal patterns associated with current *H. pylori* infection.

## 2 PATIENTS AND METHODS

**A**prospective study included 112 adult patients referred for upper endoscopy for variable indications. They all had untreated active *H. pylori* infection diagnosed by a C^13^ urea breath test (UBT). The study was carried out from March 2021 to January 2022 at Nizwa General Hospital, Ministry of Health, Sultanate of Oman. Exclusion criteria: history of any of the following; recent use of antibiotics, bismuth‐containing compounds (2 weeks), and PPIs (4 weeks), recent or acute upper gastrointestinal bleeding, gastric surgery, IM, atrophic gastritis, PUD, liver cirrhosis, portal hypertensive gastropathy, alcohol consumption, other causes of chronic gastritis (bile reflux gastritis, Crohn's disease, autoimmune gastritis, chemical gastritis, eosinophilic gastritis), infection other than *H. pylori*, achlorhydria, gastric cancer, coagulopathy, or patients without endoscopic features or signs of gastritis.

The recruited patients were assessed clinically and biochemically, then examined by upper endoscopy, initially with HD‐WLE (high definition WLE), followed by I‐scan OE 3 moods successively. The patients were randomly distributed (before endoscopy) in a 1:1 ratio (sequentially numbered) into;

Group A (nontargeted, dual biopsy): included 56 patients; two samples were taken, one from the antrum on the greater curvature, approximately 3 cm from the pyloric ring, avoiding areas of ulceration and obvious IM, and one from normal‐appearing corpus near mid‐body on the greater curvature.^9^, ^11^, ^12^ The two samples were combined and placed into one test kit.^9^

Group B (Targeted single biopsy by I‐scan OE); After careful endoscopic exam and identification by I‐scan OE, a single targeted biopsy was taken from either of the following gross or subtle abnormal mucosal patterns: (1) diffuse homogenous redness, (2) mucosal edema, (3) antral nodularity, (4) enlarged gastric fold, according to the following cascade: (1) overlapped abnormal mucosal patterns, (2) the most severe or prominent of nonoverlapped, coexistent patterns, (3) the most affected or severe part of a single pattern, (4) avoiding areas with suspected atrophic gastritis and IM with the aid of I‐san OE.

### 2.1 Gastroscopy protocol

**Figure 1:**
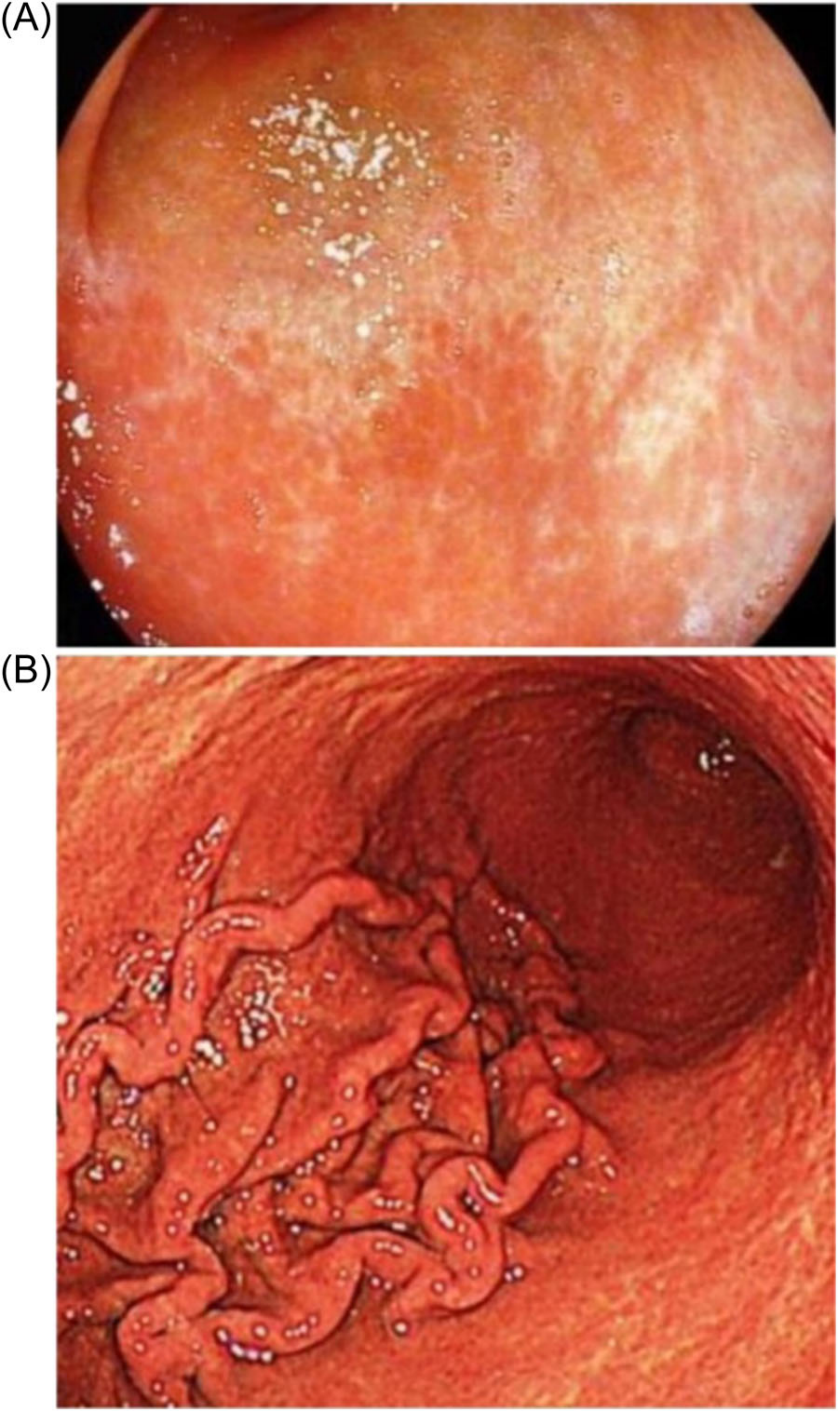
(A) Diffuse homogeneous edema (I‐scan mood 1). (B) Enlarged gastric folds (I‐scan mood 1).

**Figure 2:**
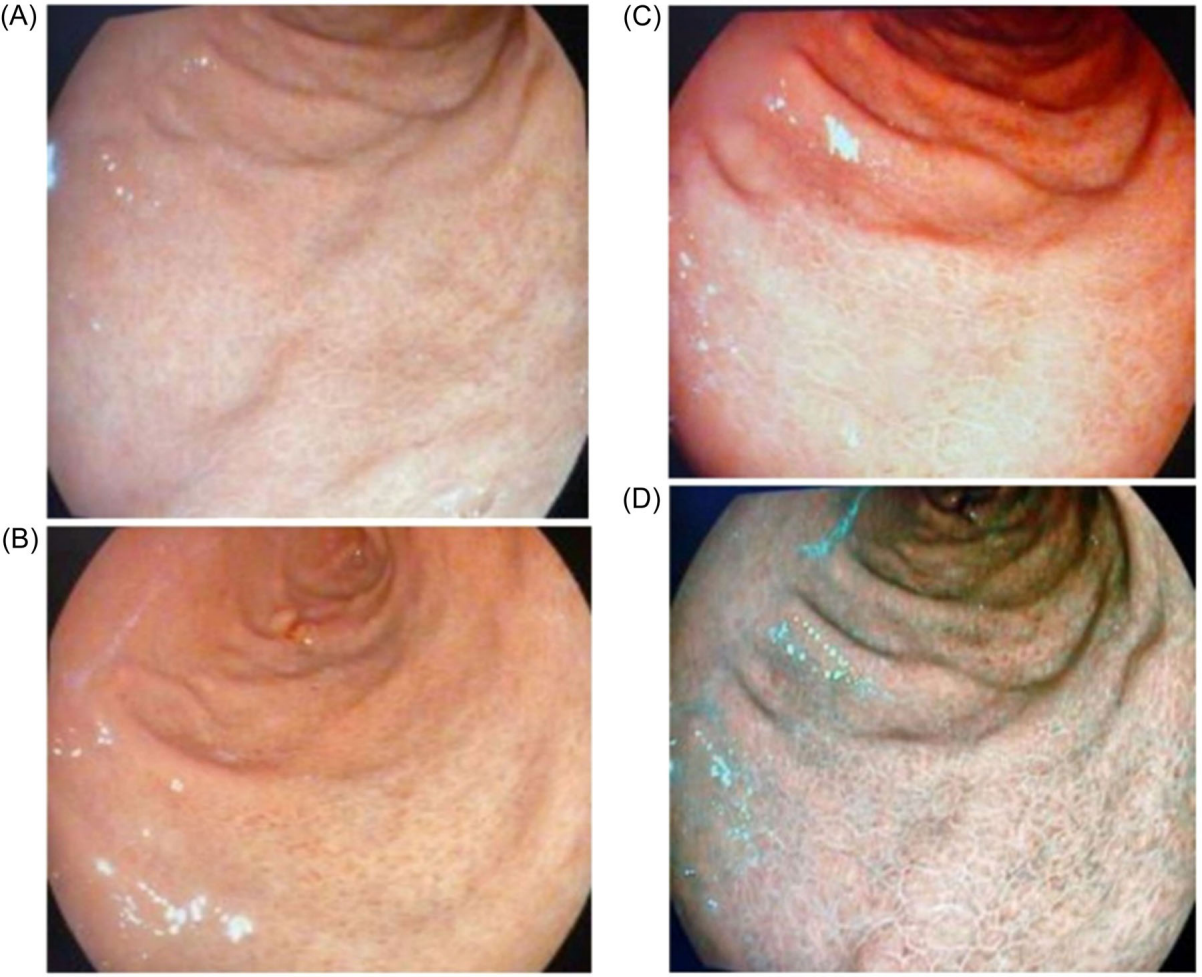
Mucosal edema; (A) white light endoscopy, (B) I‐scan mood 1, (C) I‐scan mood 2, (D) I‐scan mood 3.

**Figure 3:**
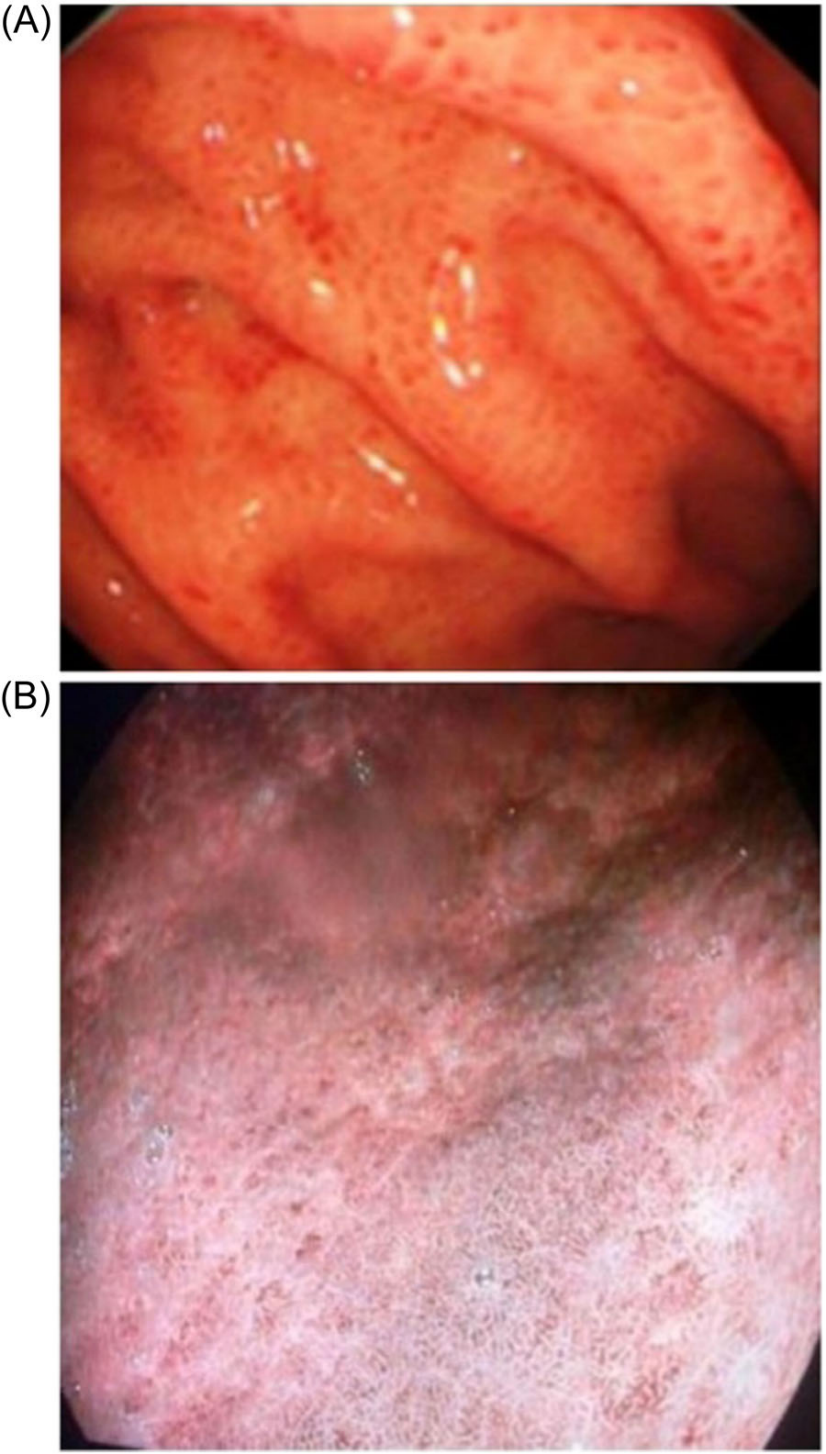
Severe mucosal edema with hyperemic red spots; (A) I‐scan mood 1, (B) I‐scan mood 3.

**Figure 4:**
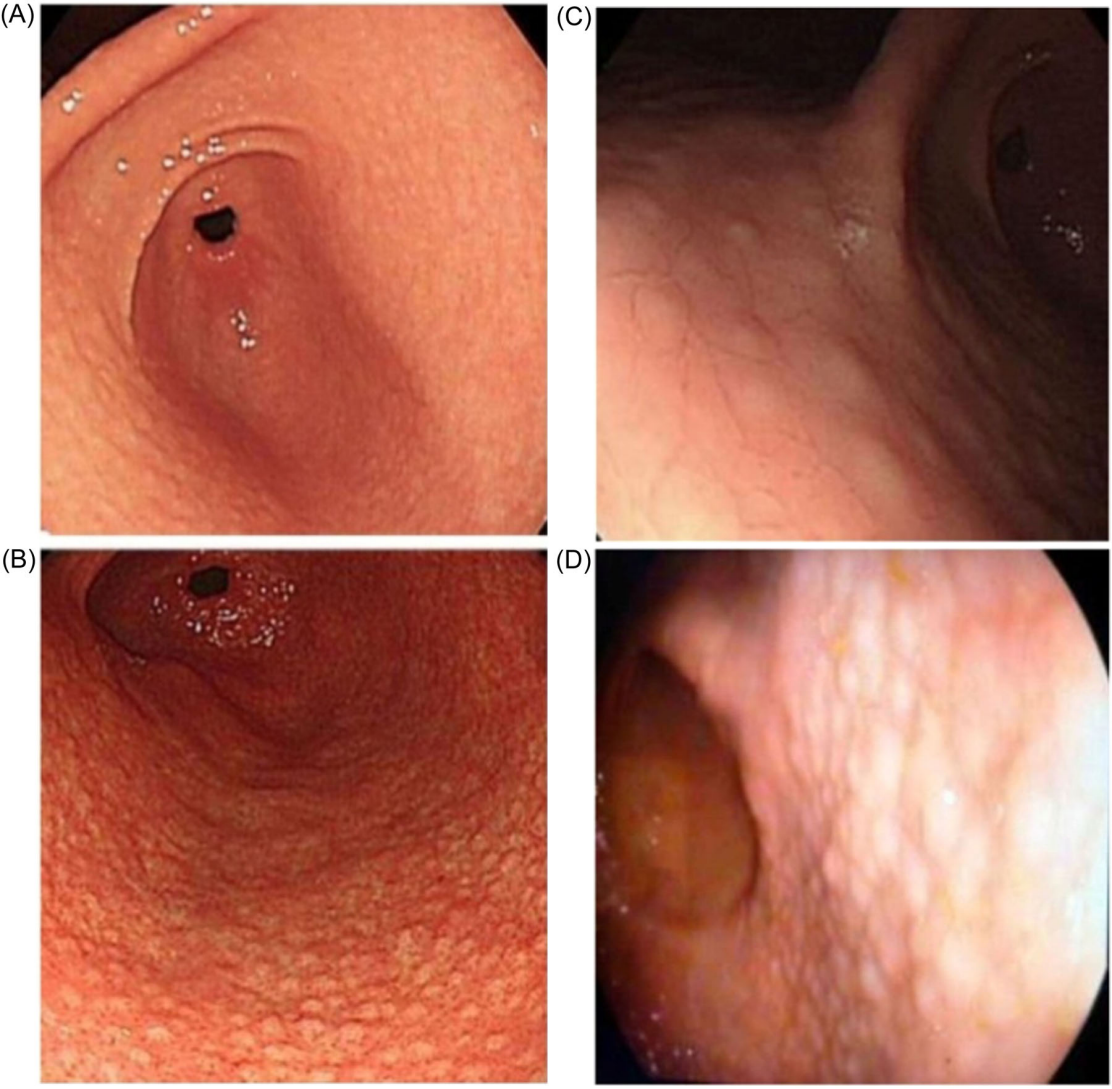
Antral nodularity; (A) White light endoscopy (WLE), (B) I‐scan mood 2, (C) close view (WLE), (D) close view (I‐scan mood 1).

**Figure 5:**
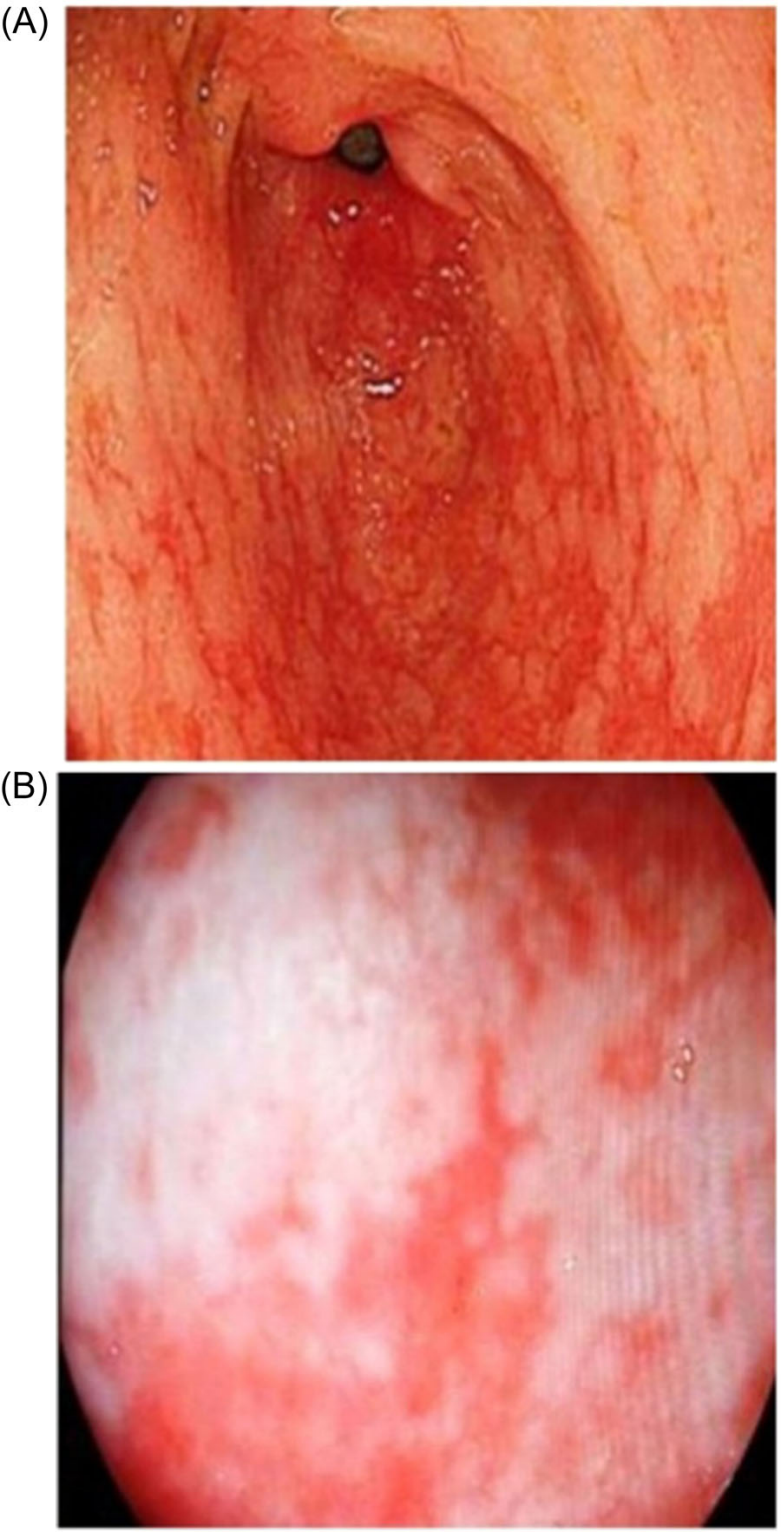
Intestinal metaplasia (IM); (A) I‐scan mood 2, (B) close view I‐scan mood 1.

All procedures were performed with PENTAX Medical EPK‐i7010, i10, high definition (nonmagnifying) endoscopy with OPTIVISTA (I‐scan OE) technology, and by a single experienced endoscopist. Patients fasted for at least 6 h. The examination was done under either pharyngeal anesthesia (xylocaine spray), sedation (midazolam ± fentanyl), or both. The stomach's antrum, corpus, and fundus were carefully examined from a medium distance after good insufflations and adequate washing, initially by WLE, then followed by I‐scan OE moods which were turned on from 1 to 3 consecutively to assess the presence of the abnormal mucosal or vascular patterns or subtle abnormalities. Biopsies were then taken according to the study protocol with the same biopsy cup size. The followings are the abnormal mucosal patterns with endoscopic description features^24^:

*Diffuse homogenous redness*: uniform redness in nonatrophic mucosa mainly located in the corpus (Figure 1A). *Enlarged gastric folds*: fold ≥5 mm, not flattened or partially flattened by insufflations (Figure 1B). *Mucosal edema*: prominent swollen area gastricae (small slightly elevated polygonal areas of gastric mucosa 1–5 mm in diameter with deep intersecting furrows^25^). It was classified as mild or severe with or without focal areas of hyperemic red dots (Figures 2 and 3). *Antral nodularity*: small miliary type resembles goose flesh in the antrum (from angle view as chicken skin appearance (Figure 4). *Atrophic gastritis*: a visible capillary network, low niveau, and yellowish pale in color as atrophic features, while diffuse redness with high mucosal height as characteristics of nonatrophic mucosa. *IM*: grayish‐white and slightly elevated plaques with a villous appearance. It is surrounded by a mix of patchy pink and pale areas of the mucosa, forming an irregularly uneven surface (Figure 5).

### 2.2 CLOrapid urease test (Kimberly‐Clark)

Its name originated from the Campylobacter‐like organism. CLOtest identifies the presence of urease enzymes, which hydrolyze urea into ammonia and bicarbonate, leading to a pH change indicated by a change in the color of phenol red from yellow (negative reading) to pink or red (positive reading). The biopsy specimens were inoculated into CLOtest kits at the same room temperature for 24 h with a reading time of the positive results at 1, 4, and 24 h. The CLOtest was discarded after 24 h (most often false positive from non‐*H. pylori* urease‐containing organisms). It should not be used to make treatment decisions.^1^, ^4^

### 2.3 I‐scan optical enhancement (OE)

I‐scan technology is a digital image‐enhanced endoscopy (IEE) technology from PENTAX Medical, based on postprocessing of reflected light with the difference of reflective properties between the normal and abnormal mucosa.^18^, ^20^ I‐scan OE is a newer technology that incorporates an additional optical filter, so it combines both digital enhancement (I‐scan) and optical enhancement (I‐scan OE). It includes three distinct modes: each mode can be accessed easily or changed by one button press on the endoscope; all patients were examined by I‐scan OE mood from 1 to 3; I‐scan mood1 (surface enhancement) for detection by enhancing the mucosal structure in a natural color tone, highlighting abnormalities, allowing a detailed inspection and topography of the mucosal surface, and better delineating lesion edges, I‐scan mood 2 (tone enhancement) for pattern characterization by enhancing the changes in vascular and mucosal structures with a color tone change, it is mainly appropriate for additional characterization of detected lesions, detecting more subtle lesions, and minute mucosal structures, I‐scan mood 3 (optical enhancement) displays the surface structures of the blood vessels, glandular ducts, and mucosal membranes in a color tone and supporting vessel characterization.^26^, ^27^

### 2.4 Data analysis

The collected data were analyzed using SPSS (IBM SPSS Statistics 28.0). Mean, standard deviation (SD), and range was used for summarizing quantitative data, *t*‐test was used for their analysis. Number and percentages were used for summarizing qualitative data, while the normal *Z*‐test was used to assess the significant difference between the two proportions. A *p*‐value of < 0.05 was considered statistically significant.

## 3 RESULTS

Table 1 shows the characteristics of the 112 patients recruited in the study [female 50, male 62, mean age 42.9 ± 14.5 (range: 17–69 years)]. There was no significant difference in the mean age of the patients between the groups. The most common indication for upper endoscopy was refractory reflux symptoms (35.7%), followed by epigastric pain (18.7%), dysphagia (10.7%), and dyspepsia (9.8%). The most common endoscopic finding was mucosal edema [mild (45.5%), severe (24.1%)], followed by homogenous redness [mild (30.4%), severe (7.1%)], enlarged gastric fold, and esophagitis (9.8%), while the least was IM (4.5%). The abnormal endoscopic mucosal patterns were nearly evenly distributed between the studied groups. The single abnormal mucosal patterns were more prevalent than multiple (more than one) abnormal patterns either overlapped or isolated (70.5% vs. 29.5%, respectively), with no significant difference between the two groups (*p* = 0.98). The most common site for biopsy in group B (targeted) was the corpus (60.7%), followed by the antrum (28.6%), then the least was the fundus (10.7%). Table 2 shows the sensitivity of the CLOtest according to the reading times of the positive results (at 1, 4, and 24 h) and the mood by which the biopsy was taken (nontargeted dual or single targeted) from the abnormal mucosal patterns. There was no significant increase in the total patients' number who had positive CLOtest readings [50% patients in group A vs. 52 patients in group B (*p* = 0.501)]. However, 78.6% of the patients in group B had a positive CLOtest at 1 h vs. 60.7% in group A (*p* = 0.047), and 17.8% of patients in group A had a positive CLOtest at 24 h (late positive results) versus 5.3% in group B (*p* = 0.032). Therefore, the reading time needed for CLOtest to turn positive was shorter if the biopsy was targeted (not random) with the aid of I‐scan OE.

**Table 1:** Clinical characteristics of the patients included in the study *n* (%)

Characteristics	Total (*n* = 112)	Group A (*n* = 56)	Group B (*n* = 56)
Age (y)			
Mean ± SD	42.9 ± 14.5	41.2 ± 13.9	44.7 ± 12.8
Range	17–69	17–68	34–69
Sex			
Female	50 (44.6)	24 (42.8)	26 (46.4)
Males	62 (55.4)	32 (57.1)	30 (53.6)
Indication for endoscopy			
Dyspepsia	11 (9.8)	5 (8.9)	6 (10.7)
Dysphagia	12 (10.7)	6 (10.7)	6 (10.7)
Epigastric pain	21 (18.7)	11 (19.6)	10 (17.8)
Refractory reflux	40 (35.7)	22 (39.3)	18 (32.1)
History of UGIB	11 (9.8)	5 (8.9)	6 (10.7)
Vomiting	9 (8)	4 (7.1)	5 (8.9)
Bariatric	8 (7.1)	3 (5.4)	5 (8.9)
Findings on endoscopy			
Mucosal edema			
Mild	51 (45.5)	22 (39.2)	29 (51.7)
Severe	27 (24.1)	12 (21.4)	15 (26.7)
Diffuse homogenous Redness			
Mild	34 (30.4)	16 (28.6)	18 (32.1)
Sever	8 (7.1)	3 (5.4)	5 (8.9)
Antral nodularity	10 (8.9)	4 (7.1)	6 (10.7)
Enlarged gastric fold	11 (9.8)	5 (8.9)	6 (10.7)
IM	5 (4.5)	2 (3.6)	3 (5.4)
Atrophy	8 (7.1)	3 (5.4)	5 (8.9)
Erosion	9 (8)	4 (7.1)	5 (8.9)
Ulcer	7 (6.3)	4 (7.1)	3 (5.4)
Esophagitis	11 (9.8)	5 (8.9)	6 (10.7)
Abnormal mucosal pattern			
Single	79 (70.5)	40 (71.4)	39 (69.6)
Multiple	33 (29.5)	16 (28.6)	17 (30.7)
Site of biopsy			
Fundus	6 (5.3)	0	6 (10.7)
Corpus	84 (75.0)	56(100)	34 (60.7)
Antrum	72 (64.3)	56 (100)	16 (28.6)

**Table 2:** Number (%) of positive CLOtest in each group according to incubation time

	No. (%) positive CLOtest		
	Group B (*n* = 56)	Group A (*n* = 56)	*p*‐value
1 h	44 (78.6)	34 (60.7)	0.047
4 h	5 (8.9)	6 (10.7)	0.748
24 h	3 (5.3)	10 (17.8)	0.032
Total	52 (92.8)	50 (89.3)	0.501

## 4 DISCUSSION

The CLOtest is a commonly used RUT for detecting *H. pylori* infection with high sensitivity; however, its false‐negative results are still apparent. Missing a site rich in *H. pylori* organisms could be because the biopsy is randomly taken or taken from a normal‐looking mucosa in the corpus or incisura as recommended^11^, ^12^ instead of targeting the abnormal mucosal patterns correlated directly with *H. pylori* load and activity.^14^ Furthermore, the early reading of the test result^3^ is a postendoscopic cause of false‐negative. I‐scan OE, which can better detect and enhance abnormal mucosal patterns than HD‐WLE,^17^ will aid to direct where the mucosa with viable and presumed high *H. pylori* load is present and overcome many causes of false‐negative results.^23^ The targeted biopsy sampling can lead to more CLOtest sensitivity, decrease false‐negative results, and shorten the time to get positive readings. All of the previous will help in accurate and early decision‐making for diagnosing and managing the *H. pylori* infection. The positive CLOtest is generally to be believed, however, a negative test requires histology or other confirmation tests with more cost and time, especially when *H. pylori* infection is likely the cause of the endoscopic finding, such as a bleeding peptic ulcer, or when a false‐negative result is suspected.^11^ Targeted biopsy sampling may help overcome such limitations and avoid extra invasive or noninvasive *H. pylori* diagnostic work‐up.

Cho et al.^13^ reported that close observation (<1 cm) of the gastric mucosal pattern by standard endoscopy could predict *H. pylori* infection. There was no need for close observation in this study, which needs more examination time, as I‐scan OE technology could detect abnormal mucosal patterns at a medium distance from the gastric mucosal surface. On the other side, magnifying endoscopy (M‐Iscan) can reveal more precisely the subtle abnormal mucosal patterns in *H. pylori* infection; however, it requires more training, time, and experience,^22^ plus it was not available at the time of the study.

Usually, obtaining a single biopsy sample, especially from the antrum, for CLOtest is associated with a high sample error and very low test sensitivity. Siddique et al. reported a very low CLOtest sensitivity (52%) with a single random biopsy from the antrum and only 4% positive CLOtest readings after 1 h.^28^ This result could be attributed in part to the presence of insufficient numbers of *H. pylori* organisms in one tissue sample, the patchy uneven distribution of the organism in the mucosa, or sampling of IM (*H. pylori* does not colonize the intestinal mucosa). Moreover, choosing UBT as the gold standard test to determine the sensitivity of the CLOtest, which is less likely to be susceptible to sampling error and may be more sensitive than histology. Thus, a biopsy‐based test, such as culture, histology, or CLOtest, may underestimate the presence of *H. pylori* in the stomach if the result is based on a single biopsy sample. In contrast, in this study, a single targeted biopsy with I‐scan OE technology produced high sensitivity and almost near to CLOtest sensitivity with four random biopsy samples taken from antrum in Siddique et al. and Lee et al., studies (92.7% vs. 96% and 74%, respectively),^28^, ^29^ this highlights the effect of using I‐ scan OE to target abnormal mucosal areas of high association with active *H. pylori* infection. Biopsies for histology were taken in this study if suspected endoscopic mucosal lesions, such as IM or atrophy. Otherwise, the UBT was the used gold standard test, which was in agreement with other studies.^28^, ^29^ In addition, the histology‐based diagnosis of *H. pylori* infection is time‐consuming, expensive, with a high risk for sample error, as previously mentioned.^30^

In this study, the single targeted biopsy‐based CLOtest achieved a high sensitivity rate and overcame the previously mentioned sample error risk; however, there was no significant increase in the sensitivity over the nontargeted dual random biopsy‐based CLOtest (92.8% vs. 89.3%). On the other side, multiple biopsies for CLOtest have a higher risk of being missed, slipped, or crushed, especially if taken by biopsy forceps simultaneously or during incubation in the CLOtest kit, with resultant false‐negative results. The nontargeted dual biopsy in this study was taken separately and then combined outside before CLOtest incubation as recommended to increase sensitivity,^9^ but this was time‐consuming.

The sensitivity of CLOtest with a single random biopsy is widely affected by the severity of gastritis with atrophy. Lan et al.^10^ reported a decrease in CLOtest sensitivity when the degree of gastritis with atrophy increased from moderate to severe (from 91% to 66%, respectively), and additional corpus biopsy resulted in only a 16.67% increase in the sensitivity. It is well known that gastric mucosal atrophy and IM are the leading causes for false‐negative CLOtest. Using I‐scan OE in targeting specific abnormal mucosal patterns and avoiding suspected areas with atrophy and IM, especially in the antrum, helped to get such higher sensitivity from a single biopsy in this study. This agrees with Dohi et al., who reported that IEE technology had improved the visibility of endoscopic findings and the accuracy of endoscopic diagnosis of IM.^31^ On the other side, Tahir et al.^3^ reported that the absence of gastritis was associated with a slightly higher rate of false‐negative CLOtest results, and the use of PPI contributed only to false‐negative CLOtest in the absence of gastritis endoscopically. This highlights the importance and relationship between targeting gastritis‐associated mucosal abnormalities and the sensitivity of CLOtest as in this study. All patients were positive for *H. pylori* by UBT; it was not possible to calculate specificity for the two groups as there were no patients without *H. pylori* infection in both groups. Although *H. pylori* infection with normal‐looking endoscopy exam is present,^28^, ^32^ with the advance of IEE, as I‐scan OE, more subtle mucosal abnormalities could still be retrieved. To my knowledge, this is the first study to report on the effect of targeted gastric biopsy for CLOtest using the I‐scan OE technology for diagnosing *H. pylori* infection.

## 5 LIMITATIONS

Subjective evaluation of the severity of mucosal abnormalities and preference of one over another in sampling and inter‐observational agreement is difficult to be standardized. It is limited to a single center. The nonmagnifying I‐scan technology, as magnifying I‐scan, could detect more subtle mucosal abnormalities, especially in apparently looking normal mucosa.

## 6 CONCLUSION

Sampling a targeted gastric biopsy with the aid of I‐scan OE technology for CLOtest significantly shortens the reading time of positive results with a high test sensitivity, allowing for early decision making and saving time.

## AUTHOR CONTRIBUTION

All the work is the sole author's (Hosam M. Dawod) effort.

## CONFLICT OF INTEREST

The author declares no conflict of interest.

## ETHICS STATEMENT

This study was obtained from the review board of Health Studies & Research Approval Committee (HSRAC), Centre of Studies & Research—Ministry Of Health, Sultanate of Oman. Informed consent was obtained from all patients recruited in the study.

## TRANSPARENCY STATEMENT

The sole author (Hosam Mohamed Dawod) affirms that this manuscript is an honest, accurate, and transparent account of the study being reported; that no important aspects of the study have been omitted; and that any discrepancies from the study as planned.

## Data Availability

The datasets used and/or analyzed during the current study are available from the corresponding author on reasonable request.
